# Prognostic value of baseline ^18^F-FDG PET/CT in patients with esophageal squamous cell carcinoma treated with definitive (chemo)radiotherapy

**DOI:** 10.1186/s13014-023-02224-5

**Published:** 2023-02-24

**Authors:** Lianshuang Xia, Xiaoxu Li, Jie Zhu, Zhaisong Gao, Ju Zhang, Guangjie Yang, Zhenguang Wang

**Affiliations:** 1 Department of Nuclear Medicine, The Affiliated Hospital of Qingdao University, Qingdao, Shandong China; 2 Department of Scientific Research Management and Foreign Affairs, The Affiliated Hospital of Qingdao University, Qingdao, Shandong China

**Keywords:** ^18^F-FDG PET/CT, Prognosis, Esophageal squamous cell carcinoma, Definitive treatment

## Abstract

**Purpose:**

To investigate the prognostic value of baseline ^18^F-FDG PET/CT in patients with esophageal squamous cell carcinoma (ESCC) treated with definitive (chemo)radiotherapy.

**Methods:**

A total of 98 ESCC patients with cTNM stage T1-4, N1-3, M0 who received definitive (chemo)radiotherapy after ^18^F-FDG PET/CT examination from December 2013 to December 2020 were retrospectively analyzed. Clinical factors included age, sex, histologic differentiation grade, tumor location, clinical stage, and treatment strategies. Parameters obtained by ^18^F-FDG PET/CT included SUV_max_ of primary tumor (SUV_Tumor_), metabolic tumor volume (MTV), total lesion glycolysis (TLG), SUV_max_ of lymph node (SUV_LN_), PET positive lymph nodes (PLNS) number, the shortest distance between the farthest PET positive lymph node and the primary tumor in three-dimensional space after the standardization of the patient BSA (SD_max(LN-T)_). Univariate and multivariate analysis was conducted by Cox proportional hazard model to explore the significant factors affecting overall survival (OS) and progression-free survival (PFS) in ESCC patients.

**Results:**

Univariate analysis showed that tumor location, SUV_Tumor_, MTV, TLG, PLNS number, SD_max (LN-T)_ were significant predictors of OS and tumor location, and clinical T stage, SUV_Tumor_, MTV, TLG, SD_max (LN-T)_ were significant predictors of PFS (all *p* < 0.1). Multivariate analysis showed that MTV and SD_max (LN-T)_ were independent prognostic factors for OS (HR = 1.018, 95% CI 1.006–1.031; *p* = 0.005; HR = 6.988, 95% CI 2.119–23.042; *p* = 0.001) and PFS (HR = 1.019, 95% CI 1.005–1.034; *p* = 0.009; HR = 5.819, 95% CI 1.921–17.628; *p* = 0.002). Combined with independent prognostic factors MTV and SD_max (LN-T)_, we can further stratify patient risk.

**Conclusions:**

Before treatment, ^18^F-FDG PET/CT has important prognostic value for patients with ESCC treated with definitive (chemo)radiotherapy. The lower the value of MTV and SD_max (LN-T)_, the better the prognosis of patients.

## Introduction

Currently, esophageal cancer is the sixth leading cause of cancer death worldwide [1]. East Asia has the highest incidence rate, with squamous cell carcinoma accounting for more than 90% [2]. The prognosis of locally advanced ESCC is poor and surgery after neoadjuvant chemoradiotherapy (nCRT) is usually the standard treatment [3]. But for patients who are not suitable for surgery or refuse surgery, definitive (chemo)radiotherapy is the main treatment [4]. At present, most operable patients are staged by the 8th American Joint Committee on Cancer (AJCC) TNM staging systems, but its guiding significance and predictive value for patients receiving non-surgical treatment are limited [5, 6]. Imaging methods such as endoscopic ultrasonography (EUS), computed tomography (CT), positron emission tomography computed tomography (PET/CT), etc. [7–10] have been widely used to evaluate the prognosis of these patients. Among them, Fluorine-18 fluorodeoxyglucose positron emission tomography/computed tomography (^18^F-FDG-PET/CT) is increasingly used in esophageal cancer. Many studies have shown that ^18^F-FDG PET/CT metabolic parameters, such as the maximum standardized uptake value (SUV_max_), metabolic tumor volume (MTV) as well as total lesion glycolysis (TLG), have important clinical value in evaluating the prognosis of baseline and non-operative esophageal cancer patients, but the results are still controversial [11–15]. The detection of clinically nodes consisting with metastases at staging procedures, including ^18^F-FDG PET/CT (that shows advantages for detecting nodal metastasis over conventional imaging) is a well-recognized prognostic predictor [16, 17], however, few studies had used the dispersal distance of PET positive lymph nodes (PLNS) as a factor to evaluate the prognosis. Through a retrospective analysis of 98 patients with ESCC treated with definitive (chemo)radiotherapy, the purpose of this study was to evaluate the prognostic role of baseline ^18^F-FDG PET/CT parameters, along with clinical data, and to determine the most important prognostic factors.

## Material and methods

### Patient selection

Patients with ESCC who had definitive (chemo)radiotherapy in our hospital between December 2013 and December 2020 were analyzed retrospectively. All patients signed an informed consent form before examination. This study has been approved by the Ethics Committee of Affiliated Hospital of Qingdao University. The inclusion criteria were (1) pathological diagnosis of esophageal squamous cell carcinoma; (2) tumor with clinical stage T1–4, N1-3, M0 according to AJCC cTNM Classification of Carcinoma of the Esophagus, eighth edition; (3) no tumor related treatment before ^18^F-FDG PET/CT examination and definitive (chemo)radiotherapy was started within 2 weeks after the examination; (4) Patients with complete clinical information and followed up for at least 12 months. The exclusion criteria were (1) surgery for esophageal cancer; (2) patients with history of previous or synchronous tumors; (3) patients received palliative or supportive treatment.

### ^18^F-FDG PET/CT acquisition

All patients were scanned with the Discovery VCT 64 PET/CT system (GE Healthcare, Milwaukee, USA). Patients were asked to fast for at least 6 h before PET/CT examination, and their blood glucose level was lower than 11.1 mmol/L. Patients were intravenously injected with ^18^F-FDG at a dose of 3.7–5.5 MBq/Kg. After resting for 60 min, the patients were scanned from the vertex to the mid-thigh level to obtain whole-body CT images. (Scanning parameters: detector coverage area, 40 mm; coverage speed, 29.46 mm/s, rack rotation time, 0.7 s; tube voltage, 120kVp; tube current, 110 mA; screw pitch, 0.516:1; visual field, 70 cm; matrix, 512 × 512; slice thickness, 3.75 mm). Whole body CT was an unenhanced co-registered CT, used for attenuation correction and anatomical localization. PET scanning was performed immediately after CT, covering the same field of view. A total of 7 ~ 9 bed images (axial vision 70 cm) were collected, and each bed was scanned for 3 min. PET image reconstruction adopts a three-dimensional (3D) ordered subset expectation maximization algorithm with 20 subsets and 2 iterations. A breath holding unenhanced chest CT scan was added and the axial chest image was reconstructed with 1.25 mm slice thickness and 1.25 mm interval.

### PET image analysis

Two experienced nuclear medicine physicians (reader 1, L. X and reader 2, X. L) independently analyzed PET/CT images, and a third physician (reader 3, G. Y) made the decisions about the disputed parts. They were all blind to prognostic information. All data were measured and recorded by the same nuclear medicine physician. With the help of the American GE Advantage Workstation 4.7 software, we use 40% SUV_max_ as the threshold to automatically outline volume of interest (VOI) of the primary tumor and PLNS by referring to the images of lesions on transverse, sagittal and coronal planes, applying a manual adjustment of tumor VOI to avoid inclusion of physiological FDG-avid surrounding structures/tissues (necrotic component may affect the results of tumor volume PET segmentation). Then the SUV_max_ of primary tumor (SUV_Tumor_), MTV of primary tumor (MTV), TLG of primary tumor (TLG) and SUV_max_ of PLNS (SUV_LN_) were obtained. TLG was defined as the product of the MTV and the SUV_mean_ within the lesion. Lymph nodes with SUVmax of 2.5 or higher were considered PLNS [18, 19]. The PLNS number of each patient was recorded. In addition, we proposed a new parameter standardized distance max of PET positive lymph node and the primary tumor [SD_max(LN-T)_]. SD_max(LN-T)_ refers to the shortest distance between the farthest PET positive lymph node and the primary tumor in three-dimensional space after the standardization of the patient body surface area (BSA), using the formula $\sqrt {\left( {{\text{weight}}\; \times \;{\text{height}}} \right)/3600 }$ [20].

### Clinical and follow-up data

Patients received definitive chemoradiotherapy or definitive radiotherapy (those who could not tolerate dCRT). Chemotherapy regimens were platinum plus paclitaxel or 5-fluorouracil. The total dose target of radiotherapy ranged from 41.4 to 66 Gy, 1.8–2.2 Gy/day, 5 days/week. Clinical factors collected included age, sex, histologic differentiation grade, tumor location, clinical stage, and treatment strategies. All patients were clinically staged with a physical examination, barium meal, esophagogastroduodenoscopy (EGD), EUS, contrast-enhanced cervical/thoracic/abdominal CT, and a whole body ^18^F-FDG PET/CT. Patients were followed up regularly in outpatient clinic or by telephone. The follow-up was arranged one month after the end of treatment, once within 3 months in the initial 2 years, once every 6 months during the third and fifth years, and once a year after 5 years until the last follow-up. Barium meal, EGD, EUS and contrast-enhanced CT were used to evaluate treatment response based on evaluation criteria in solid tumors (RECIST) Version 1.1. Progression-free survival (PFS) and overall survival (OS) were selected as the index to evaluate the prognosis. PFS was defined as the duration time from the date of PET/CT examination until the date of disease progression, death, or the last end of follow-up, and OS was defined as the time from the date of PET/CT examination to the date of death of patients due to any reason or until the last end of follow-up. The follow-up deadline was December 31, 2021.

### Statistical analysis

The normal test was carried out on the measurement data. If the data obeyed the normal distribution, it was expressed by mean ± SD, and if the data was non-normal, it was expressed by median with 5–95 percentile range. The counting data were expressed in frequency. The optimal cut-off values for MTV and SD_max(LN-T)_ as prognostic factors were determined using the median. The Cox proportional hazards hypothesis has been tested. Univariate and multivariate analyses of clinical-pathological-metabolic variables were carried out using Cox proportional hazard model. Collinearity analysis was used to eliminate interference factors. Survival was analyzed using the Kaplan–Meier method, and intergroup differences were evaluated using the log-rank test. Independent prognostic factors were combined to further stratify patient risk. Two-sided *p* value < 0.1 in univariate analysis and two-sided *p* value < 0.05 in multivariate analysis indicated that the difference is statistically significant. All statistical analyses were conducted using IBM SPSS statistical software (version 25; SPSS, Inc., Chicago, IL, USA).

## Results

### Patient and tumor characteristics

According to the predetermined inclusion and exclusion criteria, 98 patients were included in the study. All patients had FDG-avid primary tumors. Among the 98 patients, 94 were males and 4 were females. Their ages ranged from 45 to 88 years old, with an average of (63.40 ± 8.463) years old. All patients were squamous cell carcinoma, among which 39 were poorly differentiated, 52 were moderately differentiated, and 7 were well differentiated. The primary tumor was mostly located in the upper and middle esophagus (80%). Patient and tumor characteristics are summarized in Table 1.

**Table 1:** Patient and tumor characteristics (n = 98)

Characteristic	Value (%)
Age (years) (Mean ± SD)	63.40 ± 8.46
Sex	
Male	94 (96)
Female	4 (4)
Histologic differentiation grade	
Poor	39 (40)
Moderate	52 (53)
Well	7 (7)
Tumor location	
Upper	36 (37)
Middle	42 (43)
Lower	20 (20)
Clinical T stage	
T1	11 (11)
T2	27 (28)
T3	24 (24)
T4	36 (37)
Clinical N stage	
N1	31 (32)
N2	50 (51)
N3	17 (17)
AJCC Stage	
I	2 (2)
II	11 (11)
III	36 (37)
IV	49 (50)
Chemotherapy	
Yes	83 (85)
No	15 (15)
SUV_Tumor_ (Mean ± SD)	13.76 ± 5.65
MTV (cm^3^)	20.88 (11.32, 31.74)
TLG (g)	136.90 (68.02, 258.89)
SUV_LN_	6.10 (3.40, 9.97)
PLNS number	3 (2,6)
SD_max(LN-T)_(m^−1^)	0.37 (0.20,0.59)

SUV_Tumor_, SUV_max_ of primary tumor; MTV, metabolic tumor volume; TLG, total lesion glycolysis; SUV_LN_, SUV_max_ of lymph node; PLNS, PET positive lymph nodes; SD_max(LN-T)_, the shortest distance between the farthest PET positive lymph node and the primary tumor in three-dimensional space after the standardization of the patient BSA

### Survival analysis

**Fig. 1:**
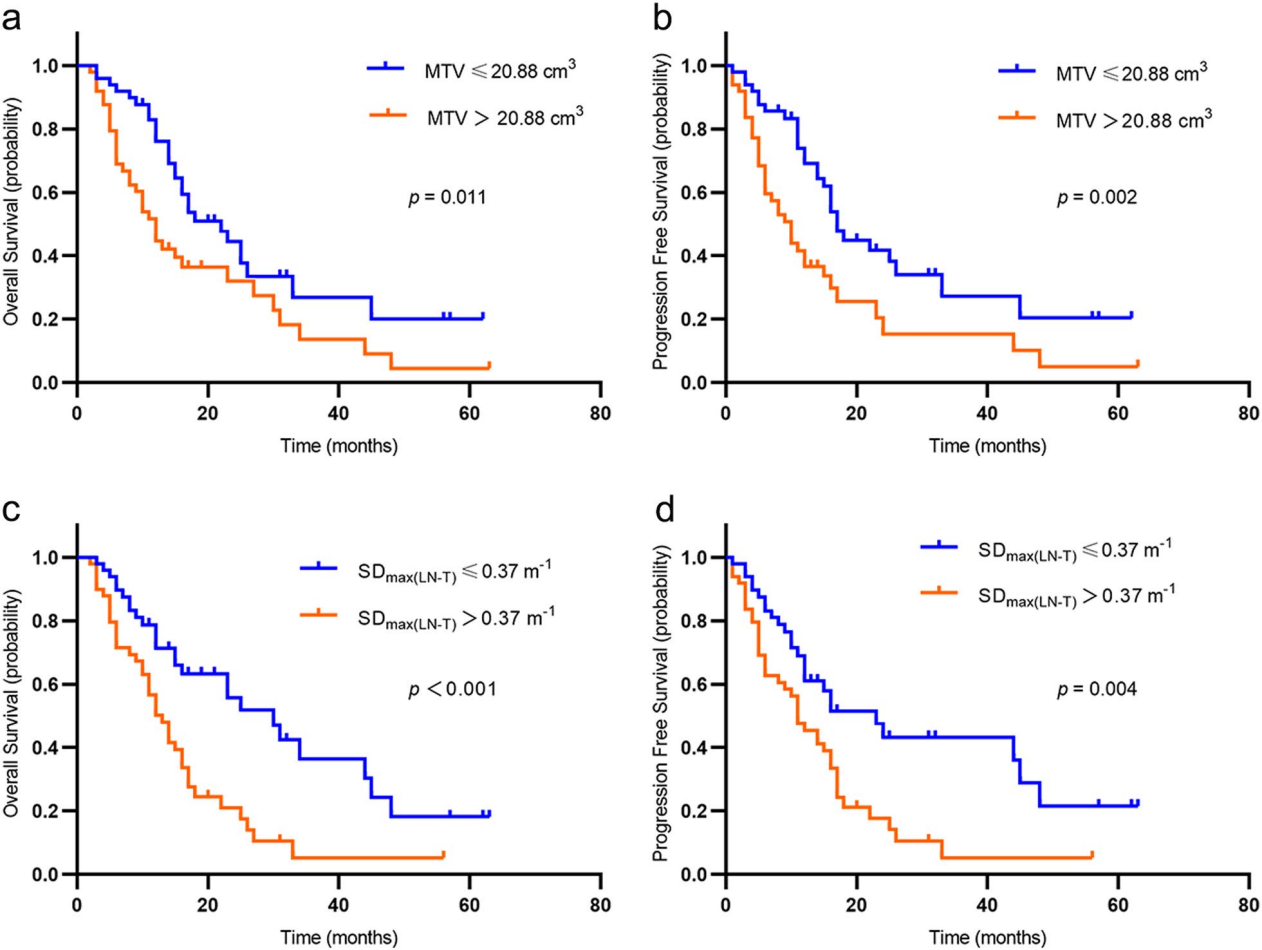
Kaplan–Meier estimates of survival functions for overall survival (OS) and progression-free survival (PFS) according to MTV (**a**, **b**) and SD_max(LN-T)_ (**c**,**d**). Log-rank* p* values are shown in the right of each figure

**Fig. 2:**
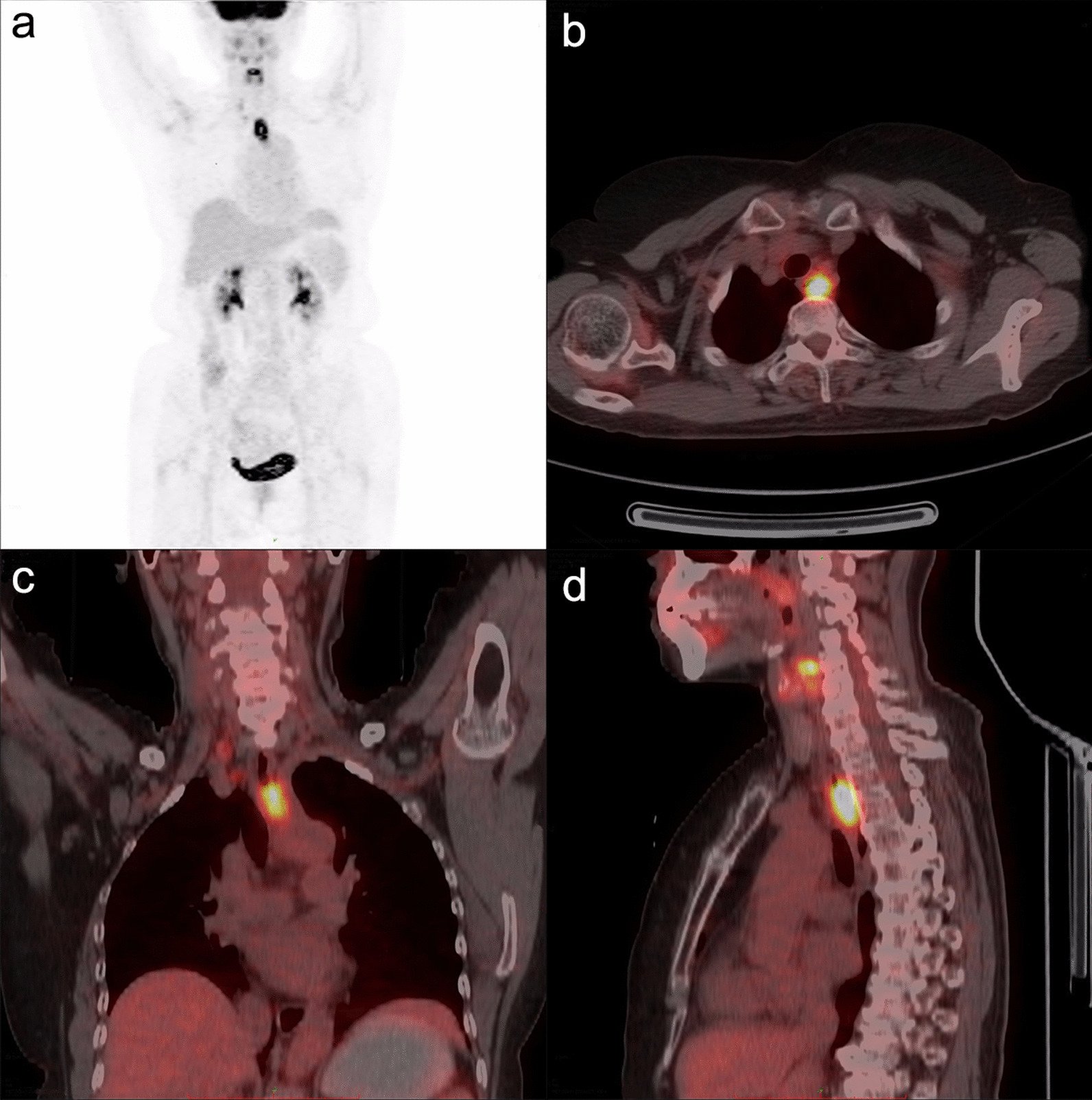
A 68-year-old female with esophageal squamous cell carcinoma. The ^18^F-FDG PET/CT fusion image showed thickening of the upper esophageal wall characterized by increased metabolism. SUV_Tumor_, MTV and TLG were 21.7, 4.69cm^3^ and 57.3 g, respectively. Hypermetabolic right cervical root and right paratracheal lymph nodes, consistent with metastases, were detected. The PLNS number was 2, SUV_LN_ and SD_max(LN-T)_ were 3.75 and 0.155 m^−1^, respectively. The patient received definitive chemoradiotherapy and was alive at the end of follow-up with an OS of 28 months

**Fig. 3:**
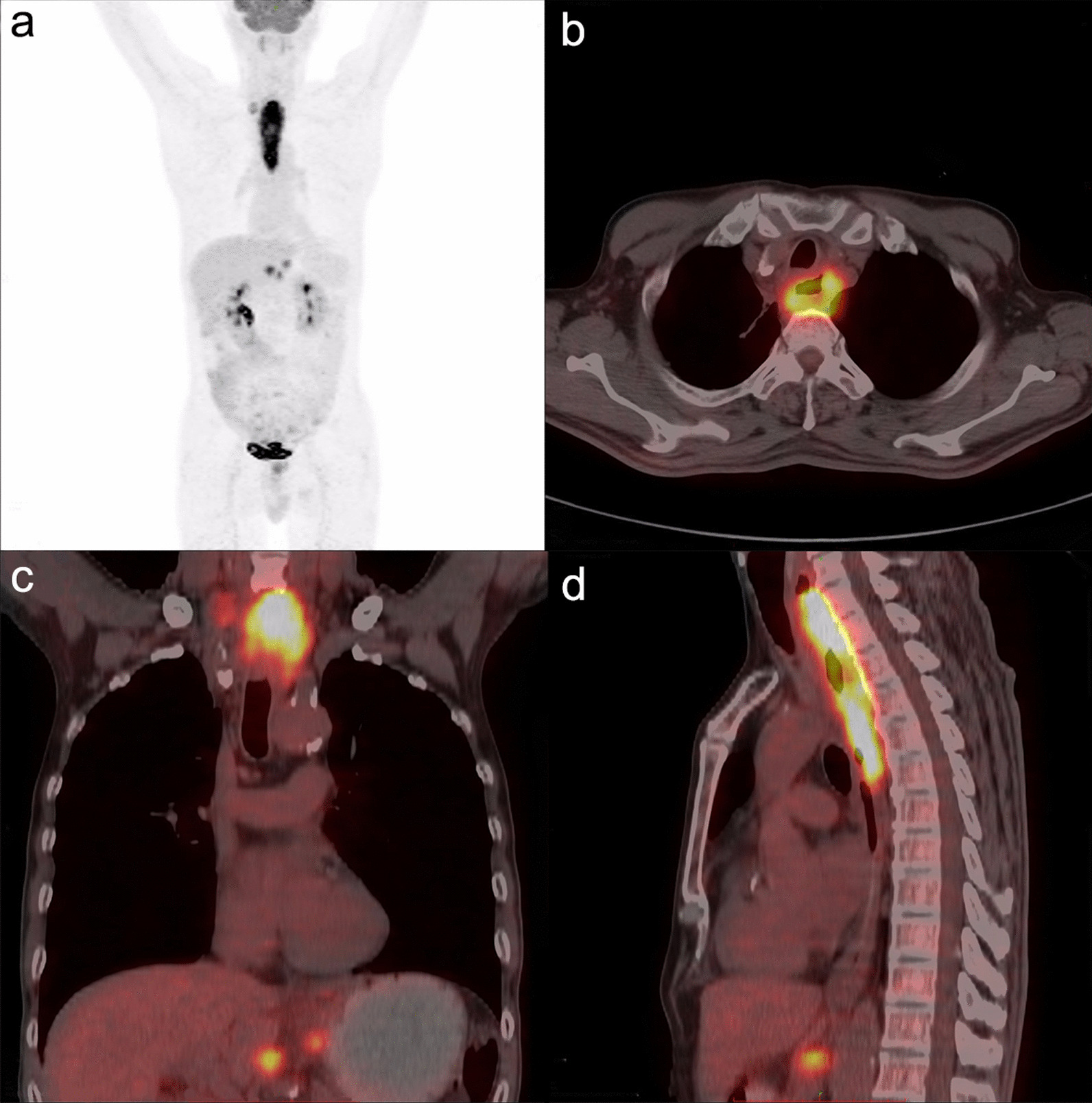
A 60-year-old male with esophageal squamous cell carcinoma. The ^18^F-FDG PET/CT fusion image showed thickening of the middle and upper esophageal wall characterized by increased metabolism. SUV_Tumor_, MTV and TLG were 17.2, 66.41 cm^3^ and 579.6 g, respectively. Lymph node metastasis in the right cervical root and hepato-gastric space, and metabolism increased. The PLNS number was 4, and the SUV_LN_ and SD_max(LN-T)_ were 8.97 and 1.03 m^−1^, respectively. The patient received definitive chemoradiotherapy and died with an OS of 4 months

The median follow-up time for the study cohort was 14 months (range 1–63 months). By the end of follow-up, 13 (13.3%) patients were alive with no disease progression. 21 (21.4%) patients were alive with local or distant progression and 64 patients (65.3%) had died. The median OS for the study cohort was 14 months, and the median PFS was 11 months. Univariate analysis showed that tumor location, SUV_Tumor_, MTV, TLG, PLNS number and SD_max (LN-T)_ were the influencing factors of OS while tumor location, Clinical T stage, SUV_Tumor_, MTV, TLG and SD_max (LN-T)_ were the influencing factors of PFS (all *p* < 0.1, Table 2). Multivariate analysis showed that only MTV and SD_max (LN-T)_ were independent prognostic factors for both OS and PFS (all *p* < 0.05, Table 3). MTV and SD_max_ were revealed as significantly negative prognostic factors for ESCC patients. Patients with higher MTV have shorter PFS (Median PFS: 8 months vs. 15 months; *p* = 0.002) and OS (Median OS: 10.5 months vs. 15.5 months; *p* = 0.011) than those with lower values, and patients with higher SD_max_ have shorter PFS (Median PFS: 11 months vs. 12 months; *p* = 0.004) and OS (Median OS: 12 months vs. 15 months; *p* < 0.001) than those with lower values (Fig. 1). Typical cases are shown in Figs. 2 and 3.

**Table 2:** Univariate Cox regression analysis in patients with ESCC

Parameters	OS			PFS		
	HR	95% CI	*p* value	HR	95% CI	*p* value
Clinical parameters						
Age (years)	0.986	0.958–1.016	0.361	0.985	0.956–1.015	0.328
Sex						
Male	1			1		
Female	0.821	0.255–2.642	0.741	0.763	0.238–2.451	0.650
Histologic differentiation grade			0.964		0.988	
Poor	1			1		
Moderate	0.956	0.648–1.412	0.822	0.977	0.581–1.643	0.931
Well	1.025	0.701–1.499	0.898	0.935	0.383–2.287	0.884
Tumor location			**0.006**		**0.011**	
Upper	1			1		
Middle	1.450	0.804–2.616	0.217	1.333	0.739–2.402	0.340
Lower	2.892	1.491–5.612	0.002	2.654	1.375–5.122	0.004
Clinical T stage			0.165		**0.082**	
T1	1			1		
T2	1.027	0.417–2.528	0.954	1.006	0.409–2.476	0.989
T3	2.050	0.851–4.939	0.109	2.163	0.894–5.236	0.087
T4	1.611	0.691–3.759	0.270	1.857	0.792–4.353	0.154
Clinical N stage			0.824		0.896	
N1	1			1		
N2	1.027	0.580–1.820	0.927	0.901	0.506–1.602	0.722
N3	1.252	0.588–2.665	0.560	1.034	0.487–2.196	0.930
AJCC Stage						
I–III	1			1		
IV	1.310	0.801–2.142	0.283	1.366	0.835–2.235	0.214
Chemotherapy						
Yes	1			1		
No	1.267	0.644–2.493	0.492	1.294	0.658–2.544	0.456
PET parameters						
SUV_Tumor_	1.043	0.999–1.089	**0.055**	1.054	1.009–1.101	**0.019**
MTV (cm^3^)	1.022	1.011–1.033	** < 0.001**	1.02	1.01–1.031	** < 0.001**
TLG (g)	1.002	1.001–1.004	** < 0.001**	1.002	1.001–1.003	** < 0.001**
SUV_LN_	1.025	0.993–1.057	0.129	1.025	0.993–1.057	0.121
PLNS number	1.063	0.993–1.138	**0.081**	1.037	0.968–1.110	0.304
SD_max(LN-T)_(m^−1^)	6.516	2.427–17.499	** < 0.001**	4.972	1.798–13.746	**0.002**

OS, overall survival; PFS, progression-free survival; HR, hazard ratio; CI, confidence interval; SUV_Tumor_, SUV_max_ of primary tumor; MTV, metabolic tumor volume; TLG, total lesion glycolysis; SUV_LN_, SUV_max_ of lymph node; PLNS, PET positive lymph nodes; SD_max(LN-T)_, the shortest distance between the farthest PET positive lymph node and the primary tumor in three-dimensional space after the standardization of the patient BSA

Values in bold indicate a significant result (*p* < 0.1)

**Table 3:** Multivariate Cox regression analysis in patients with ESCC

Parameters	OS			PFS		
	HR	95% CI	*p* value	HR	95% CI	*p* value
Tumor location			0.949			0.474
Upper	1			1		
Middle	1.091	0.585–2.036	0.784	1.014	0.545–1.885	0.965
Lower	1.133	0.496–2.588	0.767	1.501	0.691–3.258	0.305
Clinical T stage						0.183
T1	–	–		1		
T2	–	–		0.739	0.281–1.943	0.540
T3	–	–		1.492	0.543–4.103	0.438
T4	–	–		0.794	0.248–2.539	0.697
SUV_Tumor_	1.040	0.993–1.089	0.098	1.049	0.999–1.102	0.054
MTV (cm^3^)	1.018	1.006–1.031	**0.005**	1.019	1.005–1.034	**0.009**
PLNS number	1.013	0.940–1.091	0.738	–	–	
SD_max(LN-T)_	6.988	2.119–23.042	**0.001**	5.819	1.921–17.628	**0.002**

OS, overall survival; PFS, progression-free survival; HR, hazard ratio; CI, confidence interval; SUV_Tumor_, SUV_max_ of primary tumor; MTV, metabolic tumor volume; PLNS, PET positive lymph nodes; SD_max(LN-T)_, the shortest distance between the farthest PET positive lymph node and the primary tumor in three-dimensional space after the standardization of the patient BSA

Values in bold indicate a significant result (*p* < 0.05)

**Fig. 4:**
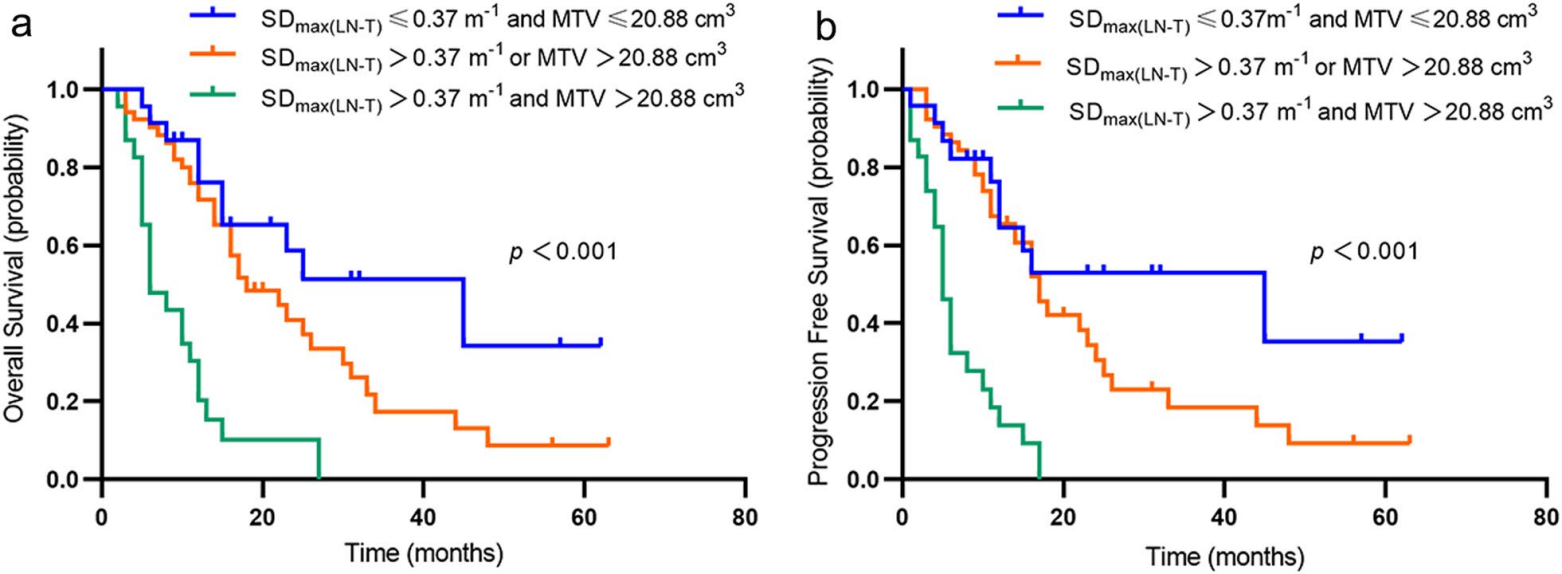
Kaplan–Meier estimates of survival functions for overall survival (OS) and progression-free survival (PFS) according to the combination of MTV and SD_max(LN-T)_ (**a**, **b**). Log-rank* p* values are shown in the right of each figure

Combined with the above two independent prognostic factors MTV and SD_max (LN-T)_, we divided the patients into three groups, group 1 (n = 23) with low MTV (≤ 20.88 cm^3^) and low SD_max (LN-T)_ (≤ 0.37 m^−1^); group 2 with either high MTV or high SD_max (LN-T)_ (n = 52), and group 3 with both high MTV and high SD_max (LN-T)_ (n = 23). We found significant differences for OS and PFS among the three groups (*p* < 0.05). Patients with high MTV and high SD_max (LN-T)_ have a worse prognosis than those with low MTV and low SD_max (LN-T)_ (Fig. 4).

## Discussion

The prognosis of esophageal cancer is poor, especially for patients in advanced stage who cannot undergo surgery, with a median survival of 16–20 months worldwide [21, 22]. The TNM staging system cannot accurately predict the prognosis of esophageal cancer patients receiving non-surgical treatment [23]. Among many imaging methods for prognosis assessment, ^18^F-FDG PET/CT examination is a promising imaging method, which can assess the systemic tumor burden through changes in glucose metabolism, providing not only anatomical information, but also reflecting the biological information of tumors. The prognostic value of primary tumor metabolic indicators such as SUV_max_, MTV and TLG for esophageal cancer have been extensively studied, but the results are still controversial [12, 24–29]. Therefore, we believe that more variables should be considered. Our study aims to evaluate the prognosis of ESCC patients treated with definitive (chemo)radiotherapy using pretreatment ^18^F-FDG PET/CT and to identify independent prognostic factors.

SUV_Tumor_ is the most commonly used metabolic parameter in PET/CT examination and many studies have investigated the prognostic value of SUV_Tumor_ in patients with esophageal cancer, However, the results are still controversial [11, 13, 30–33]. After multivariate analysis, Zhang et al. [34] found that only SUV_max_ was an independent prognostic factor for OS of patients with ESCC, while Hatt et al. [11] insisted that SUV_max_ was not. SUV_Tumor_ cannot reflect the overall characteristics of the tumor because it only represents the maximum metabolic value of the tumor. MTV is a volume measure of tumor with high glucose metabolic activity and can better characterize tumor burden. Chen et al. found that pretreatment MTV20% was a prognostic marker for patients with unresectable locally advanced esophageal cancer treated with definitive chemoradiotherapy [35]. Similarly, Sakin et al. came to a similar conclusion that pre-treatment MTV was found to be the factor associated with survival in patients treated with dCRT [36]. However, Tamandl et al. analyzed 71 patients with unresectable or metastatic esophageal carcinoma who had PET/CT examination before treatment and found that no PET parameters were associated with OS [12]. These inconsistencies might be caused by Tumor heterogeneity or the differences in treatment response rate. In our study, the prognostic value of SUV_Tumor_ was significant for OS (HR = 1.043, *p* = 0.055) and PFS (HR = 1.054, *p* = 0.019) in univariate analysis, and MTV but not SUV_Tumor_ was an independent prognostic factor for OS (HR = 1.018, *p* = 0.005) and PFS (HR = 1.019, *p* = 0.009) in multivariate analysis, which is consistent with the previous studies [24, 37, 38]. In our study, TLG was excluded from multivariate Cox regression analysis to avoid multicollinearity effect.

SD_max (LN-T)_, a new parameter proposed in our study, is defined as the closest distance from the most distant PET positive lymph node to the primary tumor, which quantifies the extent of lymph node metastasis and reflects the dissemination of the primary tumor to some extent. The prognostic value of similar distance parameters has been confirmed in lymphoma [20]. Previous studies on the extent of lymph node metastasis in esophageal cancer have shown that patients can be risk stratified according to the number of metastatic fields confirmed after surgery [39, 40], and the more metastatic fields, the shorter survival. In addition, Jimenez-Jimenez et al. [41] analyzed 56 patients with esophageal cancer and observed that if the involved lymph nodes were closer to the primary tumor, the survival rate of patients would be higher. Ielpo et al. [42] studied 64 postoperative patients with adenocarcinoma at the esophagogastric junction, and divided lymph nodes into proximal lymph node group (including cardia, lesser and large curvature and left gastric artery) and distal lymph node group (including lymph nodes from the celiac axis, common hepatic artery, lower mediastinum and tracheal bifurcation.). The results showed that the 5-year survival rate of the proximal group was better than that of the distal group (*p* < 0.005). However, the previous studies did not quantify the distance of lymph node metastasis, but only roughly distinguished the distance of lymph node metastasis. Our results are reliable because we standardize the distance by BSA, reducing individual differences. Our results showed that SD_max (LN-T)_ measured by ^18^F-FDG PET/CT was an independent prognostic factor for ESCC patients who underwent definitive (chemo)radiotherapy treatment. When stratified by SD_max (LN-T)_ > 0.37 m^−1^ and ≤ 0.37 m^−1^, OS and PFS was significantly different (*p* < 0.001; *p* = 0.004). When MTV and SD_max (LN-T)_ were combined, we discovered that patients may be further categorized. Patients with high MTV and high SD_max (LN-T)_ have a worse prognosis than those with low MTV and low SD_max (LN-T)_. In our study, of all the clinical-pathological-metabolic variables, only PET-derived parameters showed an independent prognostic effect, demonstrating the significant prognostic value of ^18^F-FDG PET/CT in ESCC patients.

This study has certain limitations: (1) This study was a retrospective study with selection bias; (2) It was a single-center study with small sample size; (3) The subjects of this study were patients with ESCC who received non-surgical treatment. It is not clear whether the results are applicable to other patients. (4) We used the medians of MTV and SD_max (LN-T)_ as the cut-off values. Despite some study limitations, our study evaluated a relatively homogeneous group of patients with ESCC who received definitive (chemo)radiotherapy.

In conclusion, in addition to the metabolic parameters of the primary tumor, nodal extent dissemination of the primary tumor also has important prognostic significance for esophageal cancer. The parameters MTV and SD_max (LN-T)_ obtained from ^18^F-FDG PET/CT before treatment of ESCC patients who received definitive (chemo)radiotherapy are independent prognostic factors, which can guide clinical risk stratification of patients, so as to develop individualized treatment plans. Those with a large MTV and SD_max (LN-T)_ value must be considered for aggressive treatment approaches and frequent follow-up. Finally, because of the lack of prospective studies, the results of this study should be validated by a larger sample and multicenter randomized prospective trials in the future.

## Data Availability

The datasets used and/or analysed during the current study are available from the corresponding author on reasonable request.

## Abbreviations

ESCC: Esophageal squamous cell carcinoma
AJCC: American joint committee on cancer
EUS: Endoscopic ultrasonography
CT: Computed tomography
PET/CT: Positron emission tomography computed tomography
LN: Lymph node
OS: Overall survival
PFS: Progression-free survival
HR: Hazard ratio
CI: Confidence interval
SUV_Tumor_: SUV_max_ of primary tumor
MTV: Metabolic tumor volume
TLG: Total lesion glycolysis
SUV_LN_: SUVmax of lymph node
PLNS: PET positive lymph nodes
SD_max(LN-T)_: The shortest distance between the farthest PET positive lymph node and the primary tumor in three-dimensional space after the standardization of the patient BSA
